# Stunting and overweight among children in Northeast Brazil: prevalence, trends (1992-2005-2015) and associated risk factors from repeated cross-sectional surveys

**DOI:** 10.1186/s12889-020-08869-1

**Published:** 2020-05-20

**Authors:** Haroldo da Silva Ferreira, Gabriela Tenório Albuquerque, Tamara Rodrigues dos Santos, Rosália de Lima Barbosa, Andressa Lima Cavalcante, Luísa Elvira Cavazzani Duarte, Monica Lopes de Assunção

**Affiliations:** 1 Faculty of Nutrition, Federal University of Alagoas, Campus A.C. Simões, BR 104 Norte - Km 96.7 - Tabuleiro do Martins, CEP, Maceió, AL 57072-970 Brazil; 2 Post-graduate Program in Nutrition, Federal University of Alagoas, Maceió, AL Brazil; 3 Post-graduate Program in Health Sciences, Federal University of Alagoas, Maceió, AL Brazil

**Keywords:** Child nutrition disorders, Protein-energy malnutrition, Obesity, Nutrition surveys, Preschool child

## Abstract

**Background:**

A study involving children from Alagoas (Northeast Brazil) revealed that, as a consequence of a drastic reduction in the prevalence of stunting between 1992 to 2005, (22.5 to 11.4%) combined with an increase in overweight prevalence (6.7 to 9.3%), the prevalence of these two conditions in 2005 was very close. If these trends were maintained, it is very likely that, at this time, the childhood overweight prevalence has already exceeded that of the stunting. However, no study is available to confirm this hypothesis. The identification of these changes is relevant to the planning and evaluation of public policies. This study aimed to investigate the prevalence, time trends and associated factors with stunting and overweight in children from Alagoas.

**Methods:**

Independent cross-sectional household surveys were conducted in 1992 (*n* = 1231), 2005 (*n* = 1381) and 2015 (*n* = 988). Data were collected from probabilistic samples of children aged 0–60 months. Stunting was defined by stature-for-age < − 2 sd and overweight by weight-for-stature > 2 sd.

**Results:**

Between 1992, 2005 and 2015, the stunting prevalence was 22.6, 11.2 and 3.2% (reduction of 85.8%), while the overweight prevalence was 6.9, 7.5 and 14.9% (increase of 115.9%), respectively. After multivariate analysis, the following positive associations with stunting were observed in 1992: age group > 24 months (28.3% vs 14.5%), mother with ≥2 children (28.8% vs 12.8%), low birth weight (28.3% vs 15.7%) and mother with low schooling (29.3% vs 7.2%). In 2015 there was a higher prevalence of stunting in males (4.2% vs 2.2%), in children < 24 months (4.6% vs 2.2%), with low birth weight (8.6% vs 3.0%) and in those who had mothers with low schooling (7.0% vs 2.6%). Regarding overweight, in 1992 there was higher prevalence for male (9.1% vs 4.7%) and in children whose mothers had ≤2 children (8.9% vs 5.8%), while in 2015 only birth weight >  4 kg was associated to overweight (27.3% vs 14.2%).

**Conclusions:**

During the analyzed period, there was a significant decrease in stunting prevalence. At the same time, a substantial increase was observed in the overweight prevalence. Currently, stunting is a problem of low magnitude, while overweight has become a worrying public health problem.

## Background

Nutritional transition is a process that has happened all over the world [1]. While this process is not complete, problems caused by the double burden of malnutrition (DBM) prevails in the population. DBM is the coexistence of nutritional deficiencies (micronutrient deficiencies, underweight, and childhood stunting and wasting) and overweight/obesity affecting countries, households, and individuals [2]. Both overweight/obesity and nutritional deficiencies in general are risk factors for adverse health consequences throughout the life-course [3].

Findings of a study involving about 130 million children, adolescents, and adults from 200 countries worldwide demonstrated that, from 1975 to 2016, the protein-calorie malnutrition prevalence in children under 5 years old has been systematically decreasing. On the other hand, the rising trends in children’s obesity have plateaued in many high-income countries, although at high levels, but continues to grow at an accelerated rate in other regions of the world. These differences are associated with the level of the nations’ economic development. Thus, in the poorest regions of the world there may be a double burden of nutritional problems [4].

Although at the individual level, stunting is not a synonym of malnutrition, in a population (epidemiological) approach, this condition is considered the most prevalent form of child malnutrition [5, 6]. In a publication by the WHO, it is reported that, in 2016, there were 22.9% or 154.8 million children under 5 years with stunting in the world [7].

Stunting is defined as impaired growth and development that children experience due to the combined effects of poor nutrition, repeated infection, and inadequate psychosocial stimulation [8]. According to the World Health Organization (WHO), factors that go beyond hunger and food availability are involved in the etiology of stunting. Child stunting is a process that occurs mostly in the first 1000 days after conception, as a result of the interaction between several characteristics, such as socioeconomic status, food intake, infections, maternal health, infectious diseases, micronutrient deficiencies and environmental conditions [7]. In this way, the adequacy of linear growth is a good indicator of children’s general health [8] and provides an accurate indicator of inequalities in human development [6]. Since stunting, once established, is difficult to be reversed, and that while a nutritional deficiency can cause stunting, there are many other non-nutritional causes, which reduces the effectiveness of nutrition-specific interventions, it calls for preventive measures articulated in multiple development sectors and requires a trans-disciplinary response [9, 10].

Childhood obesity is a public health problem that has shown increasing in prevalence in many countries around the world. This has caused great concern due to the comorbidities associated with excess body weight that occurs already during childhood (type 2 diabetes mellitus, hypertension, nonalcoholic fatty liver disease, obstructive sleep apnea, and dyslipidemia) and, especially, in adulthood, given that the obese child is more likely to become an obese adult. Only a small fraction of obese children have an underlying endocrine or genetic cause for their weight gain. Thus, the main etiology for the accumulation of fat above the level considered healthy (which defines the condition of obesity) is a positive energy balance. This is due to excessive caloric intake and/or a low pattern of physical activity, associated with a metabolic genetic predisposition for the accumulation of body fat [5, 11–13].

In Brazil, the Ministry of Health is an institution of the Federal Government that is responsible for elaborate public policies to promote the Brazilian people’s health. Thus, adequate estimates of the prevalence of the main problems that affect the population are necessary. In this aspect, there are difficulties due to the unequal pattern of economic development existing among the different states of the federation and the consequent impact on their respective epidemiological profiles [14]. The states located in the south/southeast region have high Human Development Indexes (HDI), while those belonging to the north/northeast region fall within the category of median HDI. For example, the HDI of the state of Santa Catarina is 0.774, while Alagoas has an HDI of 0.631 (these states are located in southern and in northeastern Brazil, respectively). Considered in the whole, Brazil has HDI of 0.755 (high HDI) [15].

Socioeconomic differentials are strongly reflected in the population’s nutritional status. In 1989, when there was the first large national survey involving the nutritional assessment of pre-school children from the different Brazilian states, the prevalence of stunting in Alagoas was more than seven times higher than that observed in the state of Santa Catarina (36.8% vs 4.9%) [16].

In addition to the lowest HDI and higher prevalence of stunting, Alagoas presents the worst economic and social indicators when compared to the other states of the country, such as higher rate of illiteracy and lower average family income [14]. Despite this, a study published in 2013 revealed that, as a consequence of a drastic reduction in the prevalence of stunting between 1992 to 2005 (from 22.5 to 11.4%) combined with a significant increase in overweight prevalence in the same period (from 6.7 to 9.3%), the prevalence of these two conditions among preschool children, in 2005, was very close. Maintained these trends, it is very likely that, at this time, the childhood overweight prevalence has already exceeded that of the stunting. However, no study is available to confirm this hypothesis. The identification of these changes in nutritional profiles is extremely relevant for the planning and evaluation of public policies.

The objective of this study was to describe the characteristics (prevalence, temporal trend and associated factors) of stunting and the overweight in children from a state in Northeast Brazil.

## Methods

This is a time-series study, based on three household surveys conducted in 1992, 2005 and 2015. In both, the sampling processes allowed the obtaining of representative samples of children under 5 years old in the state of Alagoas.

### Sampling procedure

#### 1992 Survey

The sample was defined using a procedure consisting of three stages. At each stage were successively and randomly selected the municipalities (20 out of a total of 102), the census tracts (eight per municipality) and households (15 per census tracts). The final sample analyzed consisted of 1228 children. According to Victora et al. [17] with this sample, it would be possible to obtain estimates of the most common health problems, with an acceptable margin of error. A detailed description of the sample design is available in another publication [18].

#### 2005 Survey

In the sample size calculation, the authors considered the following parameters: prevalence of deficit (<− 2 z scores) of height-for-age of 9.5%, a margin of error of 1.5%, confidence interval of 95% and an estimated population of 308,000 individuals. For this, 1461 children would be needed. To achieve this number, a procedure similar to that used in the 1992 survey was used (selection of 20 among 102 municipalities in Alagoas and 8 census sectors in each selected municipality), except in the 3rd stage, in which instead of eight, nine households were selected per sector. The final sample consisted of 1381 children. More details are available elsewhere [18].

#### 2015 Survey

The original study aimed to estimate the prevalence and factors associated with food insecurity in families in the state of Alagoas. Socioeconomic, demographic and anthropometric data were investigated for all individuals in a representative sample of 3366 families [14]. Specifically for this study, all children under 5 years old living in the selected households were included, excluding those with anatomical or pathological alterations that could alter the anthropometric evaluation. In the sample calculation (a posteriori) was considered: overweight as a dependent variable, with a prevalence of 9.7% [19], a universe of 328,000 children, margin of sample error of 2.5% and 1.5 for correction of the effect of the complex design. For a 95% CI, with 10% more to cover possible sample loss, 946 children would be needed. The sample size analyzed was 988 individuals. The StatCalc tool from Epi-info, version 7.2.1.0 (Centers for Disease Control and Prevention - CDC, Washington, USA), available at https://www.cdc.gov/epiinfo/index.html, was used to perform the calculations.

The sampling process was similar to those carried out in the 1992 and 2005 surveys: 1) drawing of 30 municipalities (out of a total of 102); 2) four census tracts per municipality, guaranteeing the proportion between the urban and rural areas; 3) one block in each of the census tracts, and; 4) a household in each block and the 30 consecutive homes to it. Thus, 31 families were visited. The original work was published by Costa et al. [14], in which further details can be found.

### Data collection

In all the three surveys, the data were obtained by adequately trained and supervised interviewers. All procedures and instruments were tested in a pilot study. Information was obtained in the respective households through interviews applied to mothers or guardians of the children. On that same occasion, anthropometric measurements were also obtained. Although the equipment used in each of the three surveys was not exactly the same, they were systematically calibrated and tested against a standard measure, so that this aspect certainly did not interfere with the reliability of the results.

### Dependent variables

The dependent variables were stunting, defined by stature-for-age < − 2 standard deviations (sd), and overweight, identified by weight-for-stature > 2 sd. The classification of the children’s nutritional status was performed after processing of the variables weight, age, sex and stature in the Anthro v3.2.2 software (World Health Organization - WHO, Geneva, Switzerland) to obtain the z scores of the indices stature-for-age and weight-for-stature according to the WHO anthropometric standard [20].

In 1992, body weight was measured with a Salter type portable scale (CMS PBW-235; CMS Weighing Equipment, London, England), accurate to 100 g; in 2005 a portable electronic scale was used, with a capacity of 180 kg and subdivisions of 100 g (Marte PP180®; Marte Balanças Ltda., São Paulo, Brazil); in 2015 a digital scale (Charder® MS6121R, Taichung City, Taiwan) was used, with a capacity of 250 kg and a precision of 100 g. In the three surveys, the scales were calibrated weekly against standard weight.

Children older than 24 months had their stature measured in the standing position with a vertical stadiometer, while in children aged 24 months or less the length was measured with the child in the supine position, using a horizontal anthropometric ruler. The equipment in all the surveys included a non-flexible measuring tape, accurate to 0.1 cm.

### Independent variables

Through interview following questions contained in a structured form, demographic and socioeconomic variables were investigated: sex, age range (≤ 24 months; > 24 months), birth weight (obtained through the “Child’s Health Card” as a continuous variable but analyzed as a categorical variable: normal: 2500 to 3999 g; low: < 2500 g and; high: ≥4000 g), number of people living in the household (≤4; > 4), area of residence (urban; rural), and, about mother, number of children (≤2; > 2), schooling (≤4 years; > 4 years) and age range (≤ 20; 20.1 to 39.9 and ≥ 40 years).

About the area of residence, the Brazilian Institute of Geography and Statistics (IBGE) defines an urban area as that corresponding to towns (municipal headquarters), villages (county headquarters) or isolated urban areas. All territory out of the limits of an urban area is considered as a rural area. Legally, these territorial limits are established by municipal law. In general, an urban area is characterized by higher population density, greater infrastructure of public services and an economy based on industry and commerce. In rural areas, there is lower demographic density, greater distance between households, poor infrastructure of public services and agriculture and livestock is the main economic activity [21]. As already mentioned, in the sample composition of the three surveys analyzed in the present study followed the definition used by IBGE when the establishment of census sectors in the municipalities. This definition was constant throughout the analyzed period.

### Statistical analysis

The data were typed in an independent double entry in a form generated in Epi-info, version 3.5.4. After the typing errors corrections and outliers exclusion, the data were submitted to statistical analysis with the help of Stata 12.0 software (Stata Corp., College Station, USA).

Since the variables showed adherence to the normal distribution, according to the Levene and Kolmogorov-Smirnov tests, the one-way ANOVA with Bonferroni post-hoc test was used to compare means of z scores.

The observed increases (or decreases) in the prevalence at the third survey concerning the first, were described as percentages, using the following equation: [(prevalence in 2015 - prevalence in 1992)/prevalence in 1992] × 100.

Trends analysis in the dependent variables used Poisson regression to estimate prevalence ratios (PR). Increases and decreases were defined as PR > 1 or PR < 1, respectively, and the values obtained in 1992 were used as the reference.

To identify the factors associated with stunting or with overweight in the 1992 and 2015 surveys, respectively, multivariate Poisson regression was used. The independent variables that exhibited less significance (higher *p-*value) were gradually excluded from the model (backward elimination). In the final model, only those with *p* < 0.05 remained, a condition that was assumed in all the situations of the statistical analysis to designate statistical significance. However, to avoid not neglecting the possible effect of variables that approached the statistical significance, it was designated as of marginal significance those variables that were between *p* > 0.05 and *p* ≤ 0.1.

To identify possible problems caused by multicollinearity, we examined the correlation matrix. We found that there were no predictors that showed a high correlation with any of the other predictors (multicollinearity arises when at least two highly correlated predictors are assessed simultaneously in a regression model). Additionally, the variance inflation factor (vif) test was applied, with no evidence of multicollinearity in the final models obtained.

Data analysis was performed using the svy command in Stata, since the data were from complex sampling design.

### Ethical aspects

The Research Ethics Committee of the Federal University of Alagoas approved the projects for the 2005 surveys (process # 010102 / 2003–35) and 2015 (process # 010102/0355). All the participants in the study were informed about the study objectives, its risks and benefits, and the children’s mothers or guardians signed the Free and Informed Consent Form. The use of the database of the 1992 survey was carried out with the consent of the holder of the rights of this material (Prof. Dr. Cesar Gomes Victora, from the Federal University of Pelotas, RS, Brazil).

## Results

A total of 1231 children were investigated in 1992, 1381 in 2005 and 988 in 2015. The prevalence of underweight (5.9, 3.2 and 2.2%) and wasting (1.4, 0.9 and 2.1%) at the time of these surveys were, respectively, of low magnitude. For this reason, these conditions will not be explored in the present study.

In 1992 the prevalence of stunting was 22.6%, decreasing to 11.2% in 2005 and to 3.2% in 2015 (Table 1). Considering the whole period, there was a decrease in the order of 85.8% (PR = 0.14, 95% CI: 0.10–0.20). The same trend was observed with age groups, however in different magnitudes: In 1992, the prevalence among older children was almost twice that of younger children (28.3% vs 14.6%), while in 2015 there was a reversal of this profile (2.2% vs 4.6%), but at a drastically lower level.

**Table 1:** Evolution (1992, 2005 and 2015) of the prevalence (%) and prevalence ratio (PR) for stunting and overweight by age group. Children under five years old in the state of Alagoas, Northeastern Brazil

Age (Months)	Sample			Stunting PR (95% CI)			Δ (%)	Overweight PR (95% CI)			Δ (%)
	1992 (a)	2005 (b)	2015 (c)	1992	2005	2015		1992	2005	2015	
0.0 –| 24.0	511	683	434	14.6	11.3	4.6	**− 68.5**	8.1	8.4	17.1	**111.1**
				Ref.	0.78 (0.57–1.05)	0.32 (0,20-0,51)*		Ref.	1.04 (0.70–1.54)	2.10 (1.46–3.30)*	
				**haz z score (mean ± sd)**				**whz z score (mean ± sd)**			
				−0.33 ± 1.82	−0.37 ± 1.35	0.16 ± 1.49^†^		0.34 ± 1.24^†^	0.58 ± 1.12^†^	0.79 ± 1.35^†^	
24 –| 60.0	720	698	554	28.3	11.1	2.2	**−92.2**	6.1	6.5	13.2	**116.4**
				Ref.	0,39 (0,30-0,50)*	0,08 (0,04-0,14)*		Ref.	1.07 (0.71–1.60)	2.17(1.51–3.11)*	
				**haz z score (mean ± sd)**				**whz z score (mean ± sd)**			
				−1.38 ± 1.29^†^	−0.56 ± 1.24^†^	−0.09 ± 1.08^†^		0.54 ± 0.93	0.44 ± 1.09	0.53 ± 1.36	
**TOTAL**	1.231	1.381	988	22.6	11.2	3.2	**−85.8**	6.9	7.5	14.9	**115.9**
				Ref.	0,49 (0,41–0.60)*	0,14 (0,10–0.20)*		Ref.	1.08 (0.81–1.43	2.15 (1.67–2.78)*	
				**haz z score (mean ± sd)**				**whz z score (mean ± sd)**			
				−0.94 ± 1.61^†^	−0.46 ± 1.30^†^	0.02 ± 1.28^†^		0.45 ± 1.07	0.51 ± 1.11	0.64 ± 1.36^†^	

Stunting: height-for-age (haz) < −2 standard deviations (sd);

Overweight: weight-for-height (whz) > 2 sd

Δ (%) = [(c-a)/a] × 100

* Statistically significant difference, according to the 95% confidence interval (95% CI)

^†^ Statistically significant difference, according to one-way ANOVA with Bonferroni post-hoc test

The mean z-score of the height-for-age index increased from − 0.94 ± 1.61 in 1992 to − 0.46 ± 1.35 in 2005 and to 0.02 ± 1.28 in 2015. The difference was statistically significant in all the comparisons.

Regarding overweight (Table 1), the prevalence in 1992 was 6.9%, increasing to 7.5% in 2005 and to 14.9% in 2015, representing a total increase of 115.9% (PR = 2.15, 95% CI: 1.67–2.78). Unlike the increase observed in 2015 compared to 1992, the increase observed in 2005 did not reach statistical significance. Similar results were observed comparing children younger than 24 months with those with higher ages. Considering each of the three surveys, there was no significant difference in the overweight prevalence among the children according to these age groups (*p* > 0.05). Considering the total number of children, a significant increase in the z scores of the weight-for-height index was observed only in 1992 vs 2015 comparison, which increased from 0.45 ± 1.07 to 0.64 ± 1.36.

**Fig. 1:**
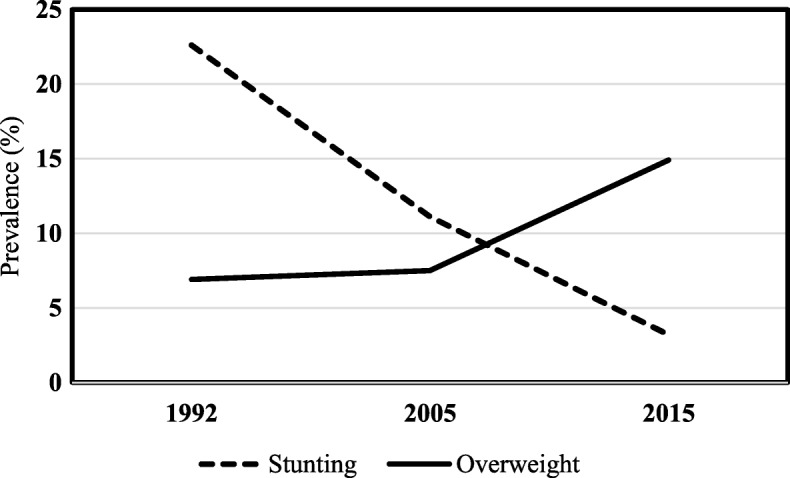
- Time trends (1992, 2005 and 2015) of the stunting and overweight prevalence. Children under five years old in the state of Alagoas, Northeastern Brazil Stunting: height-for-age < − 2 sd; Overweight: weight-for-height > 2 sd.

Comprehensively, there was an important reversal in the prevalence magnitude of the nutritional disorders analyzed, as evidenced in Fig. 1. Stunting, which was a severe public health problem in 1992, lost its epidemiological relevance for overweight in 2015, and the crossing of the trend curves that characterized this change occurred in mid-2005.

Table 2 shows the distribution of the sample socioeconomic and demographic characteristics according to the three surveys and the respective prevalence of stunting and overweight. In the period within 1992, 2005 and 2015 there was a significant reduction (*p* ≤ 0.05) in the proportion of rural residents, mothers with more than two children, children with low birth weight, children with high birth weight, mothers with lower schooling, mothers under the age of 20 and households with more than four residents.

**Table 2:** Distribution (prevalence, prevalence ratio and 95% CI) of stunting and overweight in children under five years of age, according to categories of socioeconomic and demographic variables by survey year (1992, 2005 and 2015). Alagoas, Northeast Brazil

Variable / Category	Sample distribution (%)			*P*-value (χ^**2**^)	Stunting (%)			Overweight (%)		
	1992	2005	2015		1992 Ref.	2005 RP (95% CI)	2015 RP (95% CI)	1992 Ref.	2005 RP (95% CI)	2015 RP (95% CI)
**Sex**										
Male	50.4	48.8	49.7	0.704	**23.4** Ref.	**13.1** 0.56 (0.43–0.71)	**4.2** 0.18 (0.12–0.28)	**9.1** Ref.	**7.4** 0.81 (0.56–1.17)	**15.6** 1.70 (1.22–2.36)
Female	49.6	51.2	50.3		**21.8** Ref.	**9.4** 0.43 (0.32–0.57)	**2.2** 0.10 (0.06–0.19)	**4.7** Ref.	**7.6** 1.62 (1.04–2.51)	**14.2** 3.04 (2.01–4.61)
					*p = 0.488*	*p = 0.038*	*p = 0.076*	*p = 0.002*	*p = 0.906*	*p = 0.558*
**Age group (months)**										
≤ 24	41.4	49.6	43.9	< 0.001	**14.5** Ref.	**11.3** 0.78 (0.57–1.05)	**4.6** 0.31 (0.20–0.51)	**8.1** Ref.	**8.4** 1.04 (0.70–1.54)	**17.1** 2.10 (1.46–3.03)
> 24	58.6	50.4	56.1		**28.3** Ref.	**11.1** 0.39 (0.30–0.50)	**2.2** 0.08 (0.04–0.14)	**6.1** Ref.	**6.5** 1.10 (0.71–1.60)	**13.2** 2.17 (1.51–3.10)
					*p < 0.001*	*p = 0.887*	*p = 0.032*	*p = 0.178*	*p = 0.178*	*p = 0.092*
**Low birth weight (< 2500 g)**										
Yes	9.7	6.3	6.2	0.006	**28.3** Ref.	**18.3** 0.65 (0.36–1.17)	**8.6** 0.30 (0.12–0.76)	**4.95** Ref.	**3.33** 0.67 (0.12–3.60)	**8.62** 1.74 (0.48–6.32)
No	90.3	93.7	93.8		**15.0** Ref.	**10.3** 0.69 (0.54–0.88)	**2.8** 0.18 (0.12–0.28)	**7.67** Ref.	**7.98** 1.04 (0.76–1.43)	**15.15** 1.98 (1.47–2.66)
					*p =* 0.003	*p = 0.027*	*p = 0.013*	*p = 0.381*	*p = 0.188*	*p = 0.175*
**High birth weight (≥ 4000 g)**										
Yes	14.1	8.6	7.2	< 0.001	**11.1** Ref.	**7.4** 0.66 (0.28–1.56)	**0.0** nc	**9.9** Ref.	**16.3** 1.65 (0.80–3.39)	**27.3** 2.75 (1.38–5.50)
No	85.9	91.4	92.8		**17.2** Ref.	**11.1** 0.64 (0.51–0.82)	**3.4** 0.19 (0.13–0.29)	**7.0** Ref.	**6.9** 0.98 (0.69–1.39)	**13.8** 1.97 (1.43–2.71)
					*p = 0.105*	*p = 0.233*	*p = nc*	*p = 0.282*	*p = 0.0006*	*p = 0.003*
**Mother with ≤ 2 children**										
Yes	40.5	68.1	69.5	< 0.001	**12.8** Ref.	**8.3** 0.64 (0.46–0.91)	**2.7** 0.21 (0.13–0.35)	**8.9** Ref.	**8.5** 0.96 (0.65–1.41)	**15.0** 1.69 (1.20–2.39)
No	59.5	31.9	30.5		**28.8** Ref.	**15.1** 0.52 (0.39–0.71)	**4.8** 0.17 (0.10–0.28)	**5.79** Ref.	**5.69** 0.98 (0.57–1.70)	**14.33** 2.47 (1.64–3.72)
					*p < 0.001*	*p = 0.002*	*p = 0.098*	*p = 0.045*	*p = 0.124*	*p = 0.792*
**Low maternal schooling (years of study ≤ 4)**										
Yes	69.3	46.9	20.4	< 0.001	**29.3** Ref.	**15.8** 0.54 (0.43–0.67)	**7.0** 0.24 (0.14–0.41)	**6.4** Ref.	**5.1** 0.80 (0.51–1.26)	**13.0** 2.04 (1.30–3.22)
No	30.7	53.1	79.6		**7. 2** Ref.	**7.0** 0.97 (0.60–1.54)	**2.6** 0.36 (0.20–0.65)	**8.0** Ref.	**10.0** 1.25 (0.82–1.92)	**15.5** 1.95 (1.31–2.92)
					*p < 0.001*	*p < 0.001*	*p = 0.004*	*p = 0.323*	*p = 0.002*	*p = 0.400*
**Maternal age group (years)**										
≤ 20.0	14.1	18.1	7.2	< 0.001	**16.3** Ref.	**9.9** 0.61 (0.36–1.03)	**6.1** 0.37 (0.13–1.02)	**6.7** Ref.	**7.4** 1.10 (0.52–2.31)	**12.1** 1.80 (0.76–4.31)
20.1–39.9	76.5	75.4	82.4		**22.1** Ref.	**11.0** 0.50 (0.40–0.62)	**3.3** 0.15 (0.10–0.22)	**7.2** Ref.	**7.8** 1.09 (0.79–1.50)	**15.0** 2.10 (1.57–2.81)
≥ 40.0	9.4	6.5	10.4		**32.1** Ref.	**16.7** 0.52 (0.29–0.94)	**3.2** 0.10 (0.03–0.31)	**5.3** Ref.	**4.3** 0.82 (0.21–3.24)	**16.0** 3.03 (1.21–7.60)
					*p = 0.007*	*p = 0.277*	*p = 0.502*	*p = 0.758*	*p = 0.550*	*p = 0.781*
**Area of residence**										
Urban	57.9	62.9	74.2	< 0.001	**18.2** Ref.	**9.6** 0.53 (0.41–0.68)	**3.1** 0. 17 (0. 11–0.27)	**7.5** Ref.	**8.2** 1.09 (0.77–1.54)	**14.8** 1.97 (1.42–2.73)
Rural	42.1	37.1	25.8		**28.7** Ref.	**13.8** 0.48 (0.36–0.64)	**3.5** 0.12 (0.06–0.24)	**6.1** Ref.	**6.2** 1.02 (0.62–1.68)	**15.3** 2.49 (1.63–3.82)
					*p < 0.001*	*p = 0.026*	*p = 0.764*	*p = 0.345*	*p = 0.226*	*p = 0.841*
**Number of people in the household**										
≤ 4	29.0	43.4	58.1	< 0.001	**16.5** Ref.	**9.4** 0.57 (0.40–0.80)	**2.4** 0.15 (0.08–0.26)	**7.3** Ref.	**7.9** 1.08 (0.68–1.73)	**15.9** 2.16 (1.41–3.31)
> 4	71.0	56.6	41.9		**24.9** Ref.	**12.6** 0.50 (0.40–0.63)	**4.4** 0.17 (0.11–0.28)	**6.6** Ref.	**7.1** 1.08 (0.75–1.55)	**13.5** 2.05 (1.44–2.91)
					*p = 0.002*	*p = 0.064*	*p = 0.095*	*p = 0.655*	*p = 0.571*	*p = 0.306*

In this unadjusted analysis, stunting was associated (higher prevalence) with the following conditions: Male sex (only in 2005), age group > 24 months (in 1992) and ≤ 24 months (in 2015), low birth weight (1992, 2005 and 2005), living in a domicile with more than four residents (in 1992), living in rural areas (1992 and 2005), being a son of a mother with more than two children (in 1992 and 2005) and with low schooling (in the three surveys) and age ≥ 40 years (in 1992). On the other hand, the following conditions were associated with a higher prevalence of overweight in 2015: Female sex (only in 1992), being a son of mothers with ≤2 children (only in 1992), and high birth weight (2005 and 2015).

The adjusted analysis was performed to verify which factors were independently associated with stunting and overweight in 1992 (Table 3) and 2015 (Table 4).

**Table 3:** Factors associated with stunting and with overweight among children from zero to 60 months of age, Alagoas (Northeast Brazil), 1992

Variables	Categories	Stunting^a^			Overweight^b^		
		Prevalence (%) *p-value (χ*^*2*^***)***	Crude PR (95% CI)	Adjusted PR^c^ (95% CI)	Prevalence (%) *p-value (χ*^*2*^***)***	Crude PR (95% CI)	Adjusted PR^c^ (95% CI)
Sex	Male	23.4	1	–	9.1	1	1
	Female	21.8	0.93 (0.76–1.14) ^ns^	ns	4.7	0.57 (0.37–0.87)*	0.50 (0.32–0.78)*
		*p = 0.6640*			*p = 0.0021*		
Age group (months)	0.0 –| 24.0	14.5	1	1	8.1	1	–
	24 –| 60.0	28.3	1.94 (1.53–2.47)*	1.85 (1.31–2.63)*	6.1	0.78 (0.52–1.18) ^ns^	ns
		*p < 0.0001*			*p = 0.1781*		
Birth weight (g)	Normal (2500–3999)	15.7	1	1	7.3	1	–
	Low (< 2500)	28.3	1.80 (1.21–2.67)*	1.72 (1.19–2.49)*	5.0	0.69 (0.26–1.87) ^ns^	ns
	High (≥4000)	11.1	0.71 (0.41–1.22) ^ns^	0.58 (0.33–1.01)^#^	9.9	1.28 (0.68–2.41) ^ns^	ns
		*p = 0.0048*			*p = 0.4240*		
Number of people in the household	≤ 4	16.5	1	–	7.3	1	–
	> 4	24.9	1.51 (1.16–1.97) *	ns	6.6	0.90 (0.57–1.42) ^ns^	ns
		*p = 0.0015*			*p = 0.6548*		
Residence area	Urban	18.2	1	–	7.5	1	–
	Rural	28.7	1.58 (1.28–1.94) *	ns	6.1	0.82 (0.54–1.24) ^ns^	ns
		*p < 0.0001*			*p = 0.3449*		
Mother with ≤2 children	Yes	12.8	1	1	8.9	1	–
	No	28.8	2.25 (1.73–2.92) *	1.64 (1.15–2.35)*	5.8	0.66 (0.44–1.00) *	0.66 (0.44–1.00)*
		*p < 0.0001*			*p = 0.0450*		
Low maternal schooling (years of study ≤4)	Yes	29.3	4.07 (2.74–6.03)*	3.23 (2.01–5.22)*	6.4	0.79 (0.51–1.23) ^ns^	ns
	No	7.2	1	1	8.0	1	–
		*p < 0.0001*			*p = 0.3226*		
Maternal age group (years)	≤ 20.0	16.3	0.74 (0.52–1.06)^#^	ns	6.7	0.92 (0.49–1.70) ^ns^	ns
	20.1–39.9	22.1	1	–	7.2	1	–
	≥ 40.0	32.1	1.45 (1.08–1.95)*	ns	5.3	0.74 (0.33–1.66) ^ns^	ns
		*p = 0.0073*			*p = 0.7585*		

* Statistically significant difference, according to the 95% confidence interval (95% CI)

^ns^ Non-significant difference

# 0.05 < *p* < 0.1 (marginal statistical significance)

^a^ Stunting: height-for-age < −2 sd

^b^ Overweight: weight-for-height > 2 sd

^**c**^ backward elimination process

**Table 4:** Factors associated with stunting and with overweight among children from zero to 60 months of age, Alagoas (Northeast Brazil), 2015

Variables	Categories	Stunting^a^			Overweight^b^		
		Prevalence (%) *p-value (χ*^*2*^*)*	Crude PR (95% CI)	Adjusted PR^c^ (95% CI)	Prevalence (%) *p-value (χ*^*2*^*)*	Crude PR (95% CI)	Adjusted PR^c^ (95% CI)
Sex	Male	4.2	1	1	15.6	1	–
	Female	2.2	0.53 (0.26–1.08) ^#^	0.38 (0.17–0.83)*	14.2	0.91 (0.68–1.23)	ns
		*p = 0.076*			*p = 0.558*		
Age group (months)	0.0 –| 24.0	4.6	1	1	17.1	1	1
	24 –| 60.0	2.2	0.47 (0.23–0.95)*	0.43 (0.20–0.91)*	13.2	0.77 (0.57–1.04) ^#^	0.78 (0.58–1.07)^#^
		*p = 0.031*			*p = 0.092*		
Birth weight (g)	Normal (2500–3999)	3.0	1	1	14.2	1	1
	Low (< 2500)	8.6	2.89 (1.14–7.30)*	2.99 (1.33–6.70)*	8.6	0.61 (0.26–1.43) ^ns^	0.62 (0.26–1.46)^ns^
	High (≥ 4000)	0.0	nc (p < 0.001)	nc (p < 0.001)	27.3	1.93 (1.25–2.96)*	1.91 (1.24–2.95)*
		*p = 0.018*			*p = 0.006*		
Number of people in the household	≤ 4	2.4	1	–	15.9		–
	> 4	4.4	1.78 (0.90–3.54) ^ns^	ns	13.5	0.85 (0.62–1.16) ^ns^	ns
		*p = 0.094*			*p = 0.305*		
Residence area	Urban	3.1	1	–	14.8	1	–
	Rural	3.5	1.12 (0.54–2.40) ^ns^	ns	15.3	1.04 (0.74–1.45) ^ns^	ns
		*p = 0.764*			*p = 0.841*		
Mothers with ≤2 children	Yes	2.7	1	–	15.0	1	–
	No	4.8	1.77 (0.89–3.52) ^ns^	ns	14.3	0.96 (0.68–1.33) ^ns^	ns
		*p = 0.097*			*p = 0.791*		
Low maternal schooling (years of study ≤4)	Yes	7.0	2.68 (1.35–5.34)*	3.10 (1.52–6.33)*	13.0	0.84 (0.56–1.27) ^ns^	ns
	No	2.6	1	1	15.4	1	–
		*p = 0.004*			*p = 0.399*		
Maternal age group (years)	≤ 20.0	6.1	1.82 (0.65–5.08) ^ns^		12.1	0.81 (0.41–1.58) ^ns^	
	20.1–39.9	3.3	1		15.0	1	
	≥ 40.0	3.2	0.94 (0.29–3.09) ^ns^		16.0	1.06 (0.65–1.74) ^ns^	
		*p = 0.501*			*p = 0.781*		

* Statistically significant difference, according to the 95% confidence interval (95% CI)

^ns^ Non-significant difference

# 0.05 < *p* < 0.1 (marginal statistical significance)

^a^ Stunting: height-for-age < −2 sd

^b^ Overweight: weight-for-height > 2 sd

^**c**^ backward elimination process

The following conditions were associated with stunting in 1992 (Table 3): age group with more than 24 months (28.3% vs. 14.5%, PR = 1.85, 95% CI: 1.31–2.63), low birth weight (28.3% vs. 15.7%, PR = 1.72, 95% CI: 1.19–2.49), mother with three or more children (28.8% vs. 12.8%, PR = 1.64, 95% CI: 1.15–2.35), and mother with low schooling (29.3% vs. 7.2%, RP = 3.23, 95% CI 2.01–5.22). In 2015 (Table 4), the stunting prevalence was lower in females (2.2% vs. 4.2%, PR = 0.38, 95% CI: 0.17–0.83) and in children older than 24 months (2.2% vs. 4.6%, PR = 0.43, 95% CI: 0.20–0.91). Higher prevalence was observed among children with low birth weight (8.6% vs. 3.0%, PR = 2.99, 95% CI: 1.33–6.70) and in the mothers’ sons with low schooling (7.0% vs. 2.6%, PR = 3.10; 95% CI: 1.52–6.33).

Concerning overweight, in 1992 lower prevalence (Table 3) were found for female children (4.7% vs. 9.1%, PR = 0.50, 95% CI: 0.32–0.78) and those who had mothers with three or more children (5.8% vs. 8.9%; RP = 0.66; 95% CI: 0.44–1.0). In 2015 (Table 4), only born with more than 4 kg was associated with a higher prevalence of overweight (27.3% vs. 14.2%, PR = 1.91 and 95% CI: 1.24–2.95).

## Discussion

The stunting prevalence in children under five has declined substantially in the last three decades worldwide. Concomitantly, there was an increase in overweight prevalence. This transition in the child’s nutrition profile occurred in different proportions in both developed and developing countries [22].

The results of the present study demonstrate that this process has also been occurring in Alagoas, so that during the period investigated (1992–2015), there was an 85.8% decrease in the stunting prevalence and a 115.9% increase in the overweight prevalence.

Alongside these observations, during this period, the child mortality rate in Alagoas fell from 88.7 per 1000 live births to 14.6 per 1000 live births [23]. It is very likely that the factors associated with the reduction in the number of stunted children also contributed to the decrease in the observed mortality rate, since stunting and child mortality are correlated variables [5] and both regulated, perhaps, by factors such as the social-economic-political-emotional environment.

Considering the national context, these findings occurred in parallel with the improvement of both economic and health indicators. Gross Domestic Product (GDP) per capita, which in 1996 was R$ 5219.36, increased to R$ 29,466.85 reais in 2015 (About 1104.90 and 6237.92 US dollars, respectively, on March 10, 2020) [24]. Neonatal mortality dropped from 23.6‰ live births in 1992 to 9.4‰ live births in 2015. Similarly, the mortality of children under 5 years was reduced from 57.2‰ to 15.7‰ in the same period) [25].

This considerable decline placed the prevalence of stunting in Alagoas (3.2%) at a level lower than the world estimate (22.2%) for children of the same age, as estimated by international institutions. This discrepancy can be explained by the high prevalence found in Asia and Africa, considerably raising the global average. Analyzing the data stratified by continent, it is observed that the prevalence seen in 2015 in Alagoas is still below the average in South America (7.5%), getting close to the average attributed to the United States region (2.3%) [26].

The data presented herein are consistent with the results of a recent study that compared the health indicators measured in 1990 with those measured in 2015 in Brazil and its states [27], highlighting the expressive reduction in the prevalence of child’s stunting in the period. Between 1990 and 2015, stunting prevalence dropped from 19th to 30th place in the ranking of causes of infant mortality. The study showed that inadequate feeding was the main contributor to the overall burden of disease in the country, particularly for non-communicable diseases and, because of the worrying rise in the overweight prevalence, the authors claim that it is fundamental to invest in measures to deter this increase, such as regulatory rules to tax unhealthy foods like soft-drinks and ultra-processed foods.

The last survey that evaluated children’s health in Brazil was the National Survey of Demography and Health of Children and Women, conducted in 2006 (PNDS-2006), which found that the stunting and overweight prevalence was 7.0 and 7.3%, respectively [28]. These data corroborate with our results that indicated that from 2005 onwards, there was the intersection of the trend curves of the analyzed nutritional disorders. However, although the overweight prevalence has been similar (7.3% vs 7.5%), stunting was 4.2 percentage points higher in Alagoas (7.0% vs 11.2%).

Anthropometric surveys performed on probabilistic samples of the Brazilian population of children under five in the 1970s, 1980s and 1990s indicated a systematic concentration of stunting in the Northeast region. In 1989, the National Survey on Health and Nutrition, although indicating reduction of stunting in all the Brazilian regions, showed that the decline was relatively less pronounced in this region, which had a prevalence of height-for-age deficits three times as high as that found in the Country’s Center-South regions, a fact attributed to differentials in socio-economic development and access to the public services infrastructure [29].

Even under these circumstances, the stunting prevalence in Alagoas in 2005 (11.3%) was almost twice that of the Northeast in 2006 (5.9%) [29]. However, the rapid decline in the stunting prevalence in Alagoas in the 1992–2015 period (22.6 to 3.2%) took this nutritional disorder from the level of a medium public health problem to a problem of low magnitude [30].

These changes were consistent with modifications in families’ socioeconomic conditions, such as an increase in maternal education and a decrease in the percentage of households with mothers with three or more children.

A study conducted by Lima et al. [29] corroborates the findings of the present research by indicating that the decline in stunting in northeastern Brazil was associated with changes in socioeconomic conditions of more impoverished families, which showed improvements in purchasing power, maternal education, basic sanitation and access to health care.

The period analyzed herein, in which there was a significant change in the children’s nutritional status, encompassed the governments that promoted the country’s re-democratization, implementing a series of public policies that undoubtedly contributed to the improvement of the population’s health level. During this period, several social protection programs were performed, and “Sistema Único de Saúde” (Unified Health System) was consolidated, guaranteeing free and universal access to all citizens in their demands for health care [27]. In 2002, during the United Nations General Assembly on children, Brazil committed to reducing by at least a third of stunting prevalence in children [31, 32]. The anticipated achievement of this goal was published in 2010 by the Ministry of Health, attributing it to improved household socioeconomic conditions and access to essential public services, despite regional inequalities [33]. In this aspect, Souza et al. [34] report that:

*Regarding successful food security policies, Brazil has been known worldwide for reducing food insecurity by improving food access, income generation, supporting the food production by small farmers, and enhancing food security governance including civil society organizations. Most importantly, alongside these developments, Brazil built a robust legal and institutional framework for food security, transforming the fight against hunger into a state obligation. These political and social commitments were established in a period marked by strong economic growth and reduction of unemployment. As a result, poverty and severe food insecurity were drastically reduced from 2004 to 2014 in Brazil.*

Therefore, ensuring better socioeconomic conditions for the population, along with actions that promote universal access to education and health care is a fundamental strategy in the fight against hunger and child’s stunting.

As already mentioned, the prevalence of overweight evolved in the opposite way to that of stunting, showing an upward trend during the analyzed period, going from an irrelevant situation to a worrying public health problem [30] in this population.

The rise in the childhood overweight prevalence in Alagoas in recent decades has been higher than that observed worldwide. Reports of UNICEF/WHO/World Bank demonstrated that in 27 years (1990–2017) the worldwide overweight prevalence in children under 5 years increased from 4.8 to 5.6%, that is, the overweight prevalence in Alagoas in 1992 (6.9%) exceeded that identified in 2017 globally. The prevalence found in 2015 (14.9%) in the state was similar to that found in South Africa (13.7%) in the last global report (2017). When specifically assessing South America, the prevalence in Alagoas in 2005 was similar to the current prevalence in the continent (7.7%) [22, 26].

The increase in the childhood overweight prevalence in Brazil occurred mainly in lower economic development regions (North and Northeast), especially in the states of Alagoas, Ceará, Amazonas and Amapá [35]. Also, the rate of increase in the overweight in the country was faster in the child’s population than among adults [36].

A study that analyzed the temporal trend (1989, 1996 and 2006) of overweight in Brazilian preschoolers (24 to 59 months) identified an increase of 160% in the period, with a prevalence that increased from 3% in 1989 to 7.8% in 2006 [37]. This last value was slightly higher than that found in 2005 (6.5%) in children from Alagoas of the same age group. However, the absence of more recent national surveys and involving the same age range of the present study (0 to 59 months) makes it impossible to compare the trend observed since 2005 in Alagoas with the national trend.

Among the associated factors, low birth weight was one that exhibited the most consistent results, since it was a risk factor for stunting in both 1992 and 2015 surveys. Consistent with this fact, our investigations also revealed a drop in the low birth weight prevalence during this same period, going from 9.7 to 6.2%, respectively. On the other hand, high birth weight was independently associated with overweight prevalence in 2015. This relationship was not verified in 1992, possibly due to the very low prevalence of this nutritional condition on this date. Birth weight is a variable that expresses the nutritional status and intrauterine fetal development, possibly reflecting the influence of the mother’s eating habits during pregnancy [38, 39].

As low birth weight, low maternal education was also an independent risk factor for stunting in the 1992 and 2015 surveys. In general, greater education access contributes to nutritional deficits reduction. Although in this study there was no significant association between higher education and overweight, the overweight prevalence in children observed in recent decades had a direct relationship with the level of maternal schooling [28] and therefore with better socioeconomic conditions, which is in line with the expectations for developing countries. In Brazil and other emerging countries, studies have shown a positive association between overweight prevalence in children and family socioeconomic conditions [40–43]. According to Gupta et al. [43], this association would be due to the highest foods purchasing power, with greater access to fast-foods and ultra-processed foods (high-calorie density), combined with a more sedentary lifestyle.

The number of children was also consistent as an associated factor because “mother with three or more children” was a risk factor for malnutrition in both 1992 and 2015 survey, showing that more considerable investments in education are needed to improve the population’s health standard. In contrast, mothers with up to two children was a risk factor for overweight (only in the 1992 survey). Regarding the higher stunting prevalence among children whose mothers had three or more children, it can be speculated that, under this condition, the mother would have less time to take care of her children and, additionally, there would be less per capita availability of food at home. A Chinese study revealed that being a single-child is about four times more likely to be overweight than those having siblings, leading the authors to conclude that China’s one-child policy might have contributed to its rising childhood obesity rates [44].

Being male was a risk factor for stunting in 2015 and for overweight in 1992, which is a difficult situation to explain, except for the possible relationship with the prevalence magnitude. Difference was only observed when there was a low prevalence, such as stunting in 2015 (3.2%) and overweight in 1992 (6.9%), when boys were more affected. Given this, there is a possibility that due to the small number of individuals in the affected categories, there has been a reduction in consistency in terms of statistical power. That is, if there is a differential in terms of greater susceptibility to stunting or overweight due to sex, it was not possible to demonstrate safety in this work.

For the age group, there was association only with stunting, however oppositely: in 1992 height deficit affected children older than 24 months, while in 2015 this situation was reversed, in a way that younger children were more affected. Some authors have attributed the decline in the stunting prevalence to improvements in socioeconomic conditions and greater access to essential public services such as education, health, and sanitation among the Brazilian population. Thus, it can be assumed that children older than 24 months were more exposed and dependent on environmental conditions and therefore were more affected by stunting in 1992. With the improvements established in 2015, external factors began to interfere less, so that biological factors would better explain the higher prevalence observed among younger children.

Considering the influence of social, economic and political determinants on the children’s nutritional status [45], it is evident the importance of the results published herein to establish a baseline to be used as a parameter in future evaluations, especially because of the current scenario of political transition experienced in Brazil [34, 46].

The stunting at a global level, after years of decline, increased from 2015 to 2016 due to the economic and political crises that have been occurring in many developing countries. The economic downturns impact on food security suggests a threat to food security in Brazil, as, since 2014, the country has faced a significant economic crisis along with high political instability. As demonstrated by Costa et al. [14] in a representative probabilistic sample of families from the state of Alagoas, there was a marked increase in the food insecurity prevalence during the current Brazilian crisis. In the context of this crisis, in 2016, there was a presidential impeachment, aggravating the country’s political instability. At the same time, there was a deterioration in many social indicators, such as income and unemployment. Due to inflation, there was an increase in food prices. “*As a result, the Brazilian government responded with austerity measures, which led to reduced funding for many social and food security policies*” [34].

Completing this scenario, the inaugural speech of the current President of the Republic of Brazil, which took place on the first day of 2019, was the first since the end of the military dictatorship in 1985 not to mention the need to address poverty and inequality [47]. Still in the first day of government, extinguished the National Food and Nutrition Security Council (Conselho Nacional de Segurança Alimentar e Nutricional), a channel linked to the Presidency of the Republic to dialogue with civil society and to formulate public policies to promote food security in the country. All this has caused concern to many public health professionals in Brazil, because of the real possibility that the country reappears on the UN hunger map, from which was removed in 2014.

Therefore, the current scenario in Brazil is characterized by the weakening of government policies to reduce inequality and poverty, implemented especially from the 2000s, a fact that has been causing increase in inequalities of a social, political, economic and educational nature, conditions strongly associated with food insecurity, malnutrition and deterioration of the population’s health and quality of life [48, 49], mainly of the children.

Contrary to the important advances presented by Brazil in the fight against hunger and nutritional deficiencies, the rise in the overweight prevalence was identified. This current epidemiological scenario is worrisome due to the harmful health consequences triggered by obesity. Excessive body fat in early life increases the risk of these children to become obese adults, increasing the risk of developing noncommunicable diseases such as cardiovascular disease and type 2 diabetes mellitus [50, 51]. Given this, among the six global nutrition targets established in 2012 by the World Health Assembly Resolution 65.6 to be met by 2025 is “to ensure that there is no increase in childhood overweight” [52].

A limitation of this study was not to investigate the children’s food intake and physical activity pattern in the three analyzed periods, precluding for a more in-depth analysis based on the adequacy of energy and nutrient consumption.

In contrast to this limitation, the study provides up-to-date data on the stunting and overweight prevalence in preschoolers and their temporal trends, obtained from adequately designed and children’s representative samples from a Brazilian state.

## Conclusion

During the analyzed period (1992 to 2015), there was a significant and continuous decrease in the stunting prevalence. At the same time, a substantial increase was observed in the overweight prevalence. Currently, stunting is a problem of low magnitude, while overweight has become a worrying public health problem.

Both socioeconomic and biological factors were identified as independently associated with stunting and with overweight, characterizing the etiological complexity of these conditions.

These findings should be considered when planning actions and public policies for the prevention of these nutritional disorders.

## Data Availability

The datasets used and/or analyzed during the current study are available from the corresponding author on reasonable request.

## Abbreviations

FANUT: Faculdade de Nutrição (Faculty of Nutrition).
UFAL: Universidade Federal de Alagoas (Federal University of Alagoas).
CNPq: Conselho Nacional de Desenvolvimento Científico e Tecnológico (National Council for Scientific and Technological Development).
FAPEAL: Fundação de Amparo à Pesquisa do Estado de Alagoas (Foundation for Research Support of the State of Alagoas).
HDI: Human Development Indexes
sd: Standard deviation
95% CI: 95% confidence interval
WHO: World Health Organization
PR: Prevalence ratios
haz: Height-for-age index
whz: Weight-for-height
PNDS-2006: Pesquisa Nacional de Demografia e Saúde da Criança e da Mulher (National Survey of Demography and Health of Children and Women)
UNICEF: United Nations Children’s Fund
